# The Dosimetric Comparisons of CRT, IMRT, ARC, CRT+IMRT, and CRT+ARC of Postoperative Radiotherapy in IIIA-N2 Stage Non-Small-Cell Lung Cancer Patients

**DOI:** 10.1155/2019/8989241

**Published:** 2019-03-18

**Authors:** Yanqiu Zhang, A'meng Han, Zhanzhao Fu, Shufeng Xu, Zijian Zhang

**Affiliations:** ^1^Department of Radiotherapy, First Hospital of Qinhuangdao, Qinhuangdao, Hebei 066000, China; ^2^Information Technology Center, Yanshan University, Qinhuangdao, Hebei 066004, China; ^3^Department of Respiratory, First Hospital of Qinhuangdao, Qinhuangdao, Hebei 066000, China; ^4^Central South University of Xiangya Hospital, Department of Radiation Oncology, Changsha, Hunan 410008, China

## Abstract

Currently, studies about PORT in stage IIIA-N2 NSCLC patients in recent years have mostly adopted the conformal radiation therapy (CRT) technique, while other modern techniques such as intensity modulated radiation therapy (IMRT), volumetric modulated arc therapy (VMAT, hereinafter referred to as ARC), helical tomotherapy (HT), and so forth are also developing quickly. In this paper, we intended to compare the dosimetric characteristics of CRT, IMRT, ARC, CRT+IMRT, and CRT+ARC of PORT in stage IIIA-N2 NSCLC patients. Ten patients with stage IIIA-N2 completely resected NSCLC, whom were treated by PORT in the radiotherapy department of our hospital from January 1, 2017, to January 1, 2018, were randomly selected in this study. For each patient, the CRT plan, IMRT plan, ARC plan, CRT+IMRT plan, and CRT+ARC plan were designed separately on the same set of CT images. The isodose distribution and dose-volume histogram (DVH) of the five plans were compared to determine the dosimetric parameters of the targets, OAR (organs at risk), and the normal tissue (defined as body subtracted to PTV (planning target volume), B-P). No plan had absolute dosimetry advantages than any other plans. In clinical practice, the plans could be chosen according to their dosimetry characteristics.

## 1. Introduction

There were many controversies about postoperative radiotherapy (PORT) in IIIA non-small-cell lung cancer (NSCLC) patients with pathologically confirmed N2. For one thing, local recurrence and distant metastasis were the main reasons for treatment failure of PORT in stage IIIA-N2 NSCLC patients, as the local recurrence rate and distant metastasis rate were 23%~33% and more than 50%, respectively [1]. Furthermore, stage IIIA-N2 NSCLC patients had increased local control rates and decreased recurrence rates after surgery. However, as for survival rate, different researchers have different conclusions [2–4]. To analyze reasons for the different conclusions, one reason may be a slight difference in the standard of tumor staging in different periods. Another reason may be using different treatment models in different periods, while a third reason may be due to development stage of the radiotherapy techniques used.

Since the 1990s, radiotherapy techniques developed from two-dimensional techniques to three-dimensional precise radiotherapy techniques gradually. The three-dimensional precise radiotherapy techniques mainly contain conformal radiation therapy (CRT) and intensity modulated radiation therapy (IMRT). The IMRT has fix field IMRT, volumetric modulated arc therapy (VMAT, hereinafter referred to as ARC), and helical tomotherapy (HT). The CRT, IMRT, and ARC have been used widely in most hospitals in China and each has technical advantages of their own. The CRT is relatively safe and stable with higher marginal dose of tumor while IMRT has good conformability and uniformity. However, the VMAT not only has good conformability and uniformity, but also faster treatment efficiency and fewer machine units.

The studies about PORT in stage IIIA-N2 NSCLC patients in the past have all adopted two-dimensional techniques and/or CRT techniques and seldom IMRT or VMAT techniques. In this study, we intended to compare the dosimetric characteristics of CRT, IMRT, ARC, CRT+IMRT, and CRT+ARC of PORT in stage IIIA-N2 NSCLC patients. We hoped that our study results would provide evidence for the clinical application of above techniques.

## 2. Materials and Methods

### 2.1. Patients Selection and General Information

Ten patients with stage IIIA-N2 completely resected NSCLC, treated by PORT in radiotherapy department of our hospital from January 1, 2017, to January 1, 2018, were randomly selected in this study. There were 3 women and 7 men, aged 51~69 years, with a median age of 65 years. Planning Target Volume (PTV) was 178.2~430.8cc. The general clinical data of patients is shown in Table 1.

### 2.2. Position Immobilized and CT Scans

Patients were lying on a CT bed in supine position and immobilized using thermoplastic masks. The Ge Corp CT scanner was used to simulate the positioning. The scanning thickness and spacing were both 2.5 mm. The CT images were transmitted to the ARIA server via the Varian ARIA oncology management software. Then images were received to local at treatment planning system (TPS) terminal.

### 2.3. Delineation of Targets and Organs at Risk (OARs)

On TPS, targets and the OARs were delineated by a chief physician. According to the grouping and distribution criteria of mediastinal lymph nodes proposed by IASCLC in 2009, as for left NSCLC after complete resection, the targets included bronchial stump, 2R, 2L, 4R, 4L, 5, 6, 7, 10L, and 11L lymph nodes. As for right NSCLC after complete resection, the targets included bronchial stump, 2R, 4R, 7, 10R, and 11R lymph nodes. The OARs included spinal cord, esophagus, heart and double lung.

### 2.4. Treatment Plans Design

The plans were designed by a senior physicist in Varian Corporation Eclipse TPS (Eclipse version 10.0, Varian Medical Systems, Palo Alto, CA). For each patient, five plans were designed on the same set of CT images. 6 MV X-ray was selected, and the location of the field centers was the same. The first plan was CRT plan. Five fields were chosen for the CRT plan. The angle of fields depended on the location of tumors and followed the principle of “design fields near tumors”. Conformity and homogeneity of targets were improved by adjusting fields' weight and “field in field” techniques and avoided “hot points” and “cold points” at the same time. The second plan was IMRT plan. Five fields were also chosen for the IMRT plan and field angles were as same as the CRT plan. By setting appropriate optimization conditions and repeatedly optimizing, a plan meeting the clinical requirements was finally obtained. The third plan was ARC plan. There were two partial arcs for the ARC plan and arc positions were near tumors. The angle of collimator was 10° and 350°, respectively. The optimization conditions were the same as the IMRT plan and a plan meeting clinical requirements was finally obtained by repeated optimization.

The fourth plan was CRT+IMRT plan. Two opposing fields were chosen for the CRT plan and three fields for the IMRT plan. The three fields of IMRT plan distributed nearly as equal angle in the ipsilateral lung. Then the IMRT plan optimized based on the CRT plan, the optimization conditions were as same as the ARC plan and a plan meeting the clinical requirements was finally obtained by repeated optimization. The fifth plan was CRT+ARC plan. The CRT was the same as the CRT+IMRT plan. One partial arc was chosen for the ARC plan and optimized based on the CRT plan. The angle of collimator was 10° or 350°. The optimization conditions were the same as the CRT+IMRT plan and a plan meeting clinical requirements was finally obtained by repeated optimization.

Dose calculation of all plans was performed using analytical anisotropic algorithm (AAA). The prescription of five plans for the same patient was the same as 50 Gy, and the fraction dose was 2 Gy, a total of 25 times. The dose ratio of CRT and IMRT or ARC was 1:1. According to the NCCN guideline and related research results, the dose-limiting conditions were as follows: the prescription line encompassed over 95% of the target volume; the maximum dose of the spinal cord was less than 45 Gy; the V5 of the esophagus was less than 50%; the V30 and V40 of the heart were less than 40% and 30%, respectively; and the normal doses of normal lung volume V5, V20, and V30 were less than 60%, 25%, 20%, and 15 Gy, respectively.

### 2.5. Evaluation Methods

The isodose distribution and dose-volume histogram (DVH) of the five plans were compared to determine the dosimetric parameters of the targets, OARs, and the normal tissue (defined as Body subtracted to PTV, B-P). The targets were evaluated by the minimum dose, the maximum dose, the median dose, Homogeneity index (HI), Conformity index (CI), and the volume of the targets which were irradiated by higher than 105% prescription dose V105. HI and CI were calculated using the following equations:

(1) HI=D2−D98Dp×100%;CI=Vt,refVt×Vt,refVref

*D*_2_ and *D*_98_ refer to the maximum dose encompassing 2% and 98% of PTV, respectively, *D*_*p*_ is the prescription dose. *V*_*t*_ is the volume of PTV, *V*_*t*,*ref*_ is the volume of PTV covered by reference isodose line, and *V*_*ref*_ is the volume of the body covered by reference isodose line.

The following parameters were used to evaluate the protection of OARs: the maximum dose of spinal cord; the V_50_ and maximum dose of esophagus; V_20_, V_30_, V_40_, V_45_, and average dose of heart; V_5_, V_13_, V_20_, V_30_, and average dose of double lung. The Vx here refers to the percentage of tissue exposed to X and Gy to the total volume. The percentage of B-P exposure to 5~50 Gy volume and B-P volume, V_5~50_. In addition, the number of machine units (MU) for the five plans was also evaluated.

## 3. Statistical Analysis

SPSS 22 software package was used for statistical description and analysis of the data. The paired t test was performed to test comparisons between two plans. A 2-tailed P-value of < 0.05 was considered statistically significant.

## 4. Results

### 4.1. CRT Plan versus IMRT Plan

As shown in Table 2, the minimum dose of PTV in the CRT pan was better than the IMRT plan, and the difference was statistically significant, *t* = 6.698, *p* = 0.001. However, the Conformity index and V_105_ (%) of PTV in the CRT plan were worse than the IMRT plan, and the differences were statistically significant, *t* = −5.367, 2.359, *p* = 0.001, 0.043. The other dosimetric parameters of PTV in the CRT plan had no statistical differences compared with the IMRT plan.

As for OARs, V_50_ (%) of esophagus, V_30_ (%) of heart, and the mean dose of normal lung in the CRT plan had no statistical differences compared with the IMRT plan. The maximum dose of spinal cord in the CRT plan was higher than the IMRT plan, and the difference was statistically significant, *t* = 7.034, *p* = 0.001. The maximum dose of esophagus was lower than the IMRT plan, and the difference was statistically significant, *t* = −3.813, *p* = 0.004. The V_20_ (%), V_40_ (%), V_45_ (%), and mean dose of heart in the CRT plan were all higher than the IMRT plan, and the differences were statistically significant, *t* = 5.382, 2.864,2.832, 4.913, *p* = 0.001, 0.019,0.020, 0.001. The V_5_ (%), V_10_ (%), V_13_ (%), V_20_ (%), and V_30_ (%) of normal lung in the CRT plan were all higher than the IMRT plan, and the differences were statistically significant, *t* = 6.456, 2.653, 4.636,5.982,6.771, *p* = 0.001, 0.026, 0.001, 0.001, 0.001*;* see Table 2 for details.

As for B-P, the V_5~50_ of B-P in the CRT plan were all higher than the IMRT plan, and the differences were not statistically significant for V_5_, V_25_, and V_30_, but the differences were statistically significant for V_10_, V_15_, V_20_, V_35_, V_40_, V_45_, V_50_,* t=2.347, 2.870, 2.363, 2.317, 3.181, 2.957, 2.771, p=0.044,0.018,0.042,0.046,0.011,0.016,0.022; *see Table 2 for details.

### 4.2. CRT Plan versus ARC Plan

As shown in Table 3, the median dose, the Conformity index, and V_105_ (%) of PTV in the CRT pan were worse than the ARC plan, and the differences were statistically significant,* t=-4.433, -15.148, 2.766, p=0.002, 0.001, 0.022. *The other dosimetric parameters of PTV in the CRT plan had no statistical differences compared with the ARC plan.

As for OARs, the maximum dose of spinal cord in the CRT plan was higher than the ARC plan, and the difference was statistically significant,* t=5.516, p=0.001. *The V_50_ (%) of esophagus was lower than the ARC plan, and the difference was statistically significant,* t=-4.286, p=0.002.* But the maximum dose of esophagus in the CRT plan had no statistical differences compared with the ARC plan. The V_20_ (%), V_30_ (%), V_40_ (%), V_45_ (%), and mean dose of heart in the CRT plan were all higher than the ARC plan, and the differences were statistically significant,* t=3.369, 2.393, 2.295, 2.278, 3.129, p=0.008, 0.040, 0.047, 0.049, 0.012. *The V_5_ (%), V_10_ (%), and mean dose of normal lung in the CRT plan had no statistical differences compared with the ARC plan. However, the V_13_ (%), V_20_ (%), and V_30_ (%) of normal lung in the CRT plan were all higher than the ARC plan, and the differences were statistically significant,* t=3.860, 4.551, 5.244, p=0.004, 0.001, 0.001; *see Table 3 for details.

As for B-P, the V_5~50_ of B-P in the CRT plan were all higher than the ARC plan, and the differences were not statistically significant for V_5_, and V_10_, but the differences were statistically significant for V_15_, V_20_, V_30_, V_35_, V_40_, V_45_, V_50_,* t=4.121, 4.861, 4.142, 2.774, 2.532, 3.244, 2.999, 3.748, p=0.003, 0.001, 0.003, 0.022, 0.032, 0.010, 0.015, 0.005; *see Table 3 for details.

### 4.3. CRT Plan versus CRT+IMRT Plan

As shown in Table 4, the median dose and the Conformity index of PTV in the CRT pan were worse than the CRT+IMRT plan, and the differences were statistically significant,* t=-2.604, -7.910, p=0.001, 0.015. *The other dosimetric parameters of PTV in the CRT plan had no statistical differences compared with the IMRT plan.

As for OARs, the maximum dose of spinal cord in the CRT plan had no statistical difference compared with the CRT+IMRT plan. The maximum dose of esophagus was lower in the CRT plan than the CRT+IMRT plan, and the difference was statistically significant,* t=-2.739, p=0.023.* But the V_50_ (%) of esophagus in the CRT plan had no statistical difference compared with the CRT+IMRT plan. The V_20_ (%), V_40_ (%), V_45_ (%), and mean dose of heart in the CRT plan were all higher than the CRT+IMRT plan, and the differences were statistically significant,* t=3.369, 2.393, 2.295, 2.278, 3.129, p=0.008, 0.040, 0.047, 0.049, 0.012. *But the V_30_ (%) of heart in the CRT plan had no statistical difference compared with the CRT+IMRT plan. The V_5_ (%), V_10_ (%),V_13_ (%), and V_20_ (%) of normal lung in the CRT plan were all higher in the CRT+IMRT plan, and the differences were statistically significant,* t=5.435, 3.342, 5.082, 5.153, p=0.001, 0.009, 0.001, 0.001*. However, the mean dose and V_30_ (%) of normal lung in the CRT plan had no statistical differences compared with the CRT+IMRT plan*; *see Table 4 for details.

As for B-P, the V_5~50_ of B-P in the CRT plan had no statistical differences compared with the CRT+IMRT plan*; *see Table 4 for details.

### 4.4. CRT Plan versus CRT+ARC Plan

As shown in Table 5, the median dose and the Conformity index of PTV in the CRT plan were worse than the CRT+ARC plan, and the differences were statistically significant,* t=-2.891, -12.227, p=0.001, 0.018. *The other dosimetric parameters of PTV in the CRT plan had no statistical differences compared with the CRT+ARC plan.

As for OARs, the maximum dose of spinal cord in the CRT plan had no statistical differences compared with the CRT+ARC plan. The maximum dose and the V_50_ (%) of esophagus in the CRT plan was lower than the CRT+ARC plan, and the differences were statistically significant,* t=-2.739, -2.911, p=0.023,0.017.* The V_20_ (%), V_40_ (%), and mean dose of heart in the CRT plan were all higher than the CRT+ARC plan, and the differences were statistically significant,* t=2.933, 2.310, 2.332, p=0.017, 0.046, 0.045*. But the V_30_ (%) of heart in the CRT plan was lower than the CRT+ARC plan, and the differences were statistically significant,* t=-3.529, p=0.006. *The V_45_ (%) of heart in the CRT plan had no statistical difference compared with the CRT+ARC plan. The V_5_ (%), V_10_ (%), V_13_ (%), V_20_ (%), and V20 (%) of normal lung in the CRT plan were all higher than the CRT+ARC plan, and the differences were statistically significant,* t=3.114, 6.330, 6.673, 6.319, 2.494, p=0.012, 0.001, 0.001, 0.001, 0.034*. However, the mean dose of normal lung in the CRT plan had no statistical differences compared with the CRT+ARC plan*; *see Table 5 for details.

As for B-P, the V_15_ and V_20_ of B-P in the CRT plan were higher than the CRT+ARC plan, and the differences were statistically significant,* t=3.719, 3.261, p=0.005, 0.001*. The other dosimetric parameters of B-P in the CRT plan had no statistical differences compared with the CRT+ARC plan*; *see Table 5 for details.

### 4.5. IMRT Plan versus ARC Plan

As shown in Table 6, the minimum dose, the Conformity index, and the Homogeneity index of PTV in the IMRT pan were worse than the ARC plan, and the differences were statistically significant,* t=-5.513, -5.381, 4.768, p=0.001, 0.001, 0.001. *The other dosimetric parameters of PTV in the CRT plan had no statistical differences compared with the ARC plan.

As for OARs, the maximum dose of spinal cord, the maximum dose, and the V_50_ (%) of esophagus in the IMRT plan had no statistical differences compared with the ARC plan. The V_20_ (%) and V_30_ (%) of heart in the IMRT plan were all higher than the ARC plan, and the differences were statistically significant,* t=2.423, 2.391, p=0.038, 0.040*. The V_40_ (%), the V_45_ (%), and the mean dose of heart in the IMRT plan had no statistical differences compared with the ARC plan. The V_5_ (%) and V_10_ (%) of normal lung in the IMRT plan were lower than the ARC plan, and the differences were statistically significant,* t=-5.309, -2.697, p=0.001, 0.025*. However, The V_20_ (%) of normal lung in the IMRT plan were higher than the ARC plan, and the difference was statistically significant,* t=2.529, p=0.032. *The V_13_ (%), V_30_ (%), and the mean dose of normal lung in the IMRT plan had no statistical differences compared with the ARC plan*; *see Table 6 for details.

As for B-P, the V_5_ and V_10_ of B-P in the IMRT plan were lower than the ARC plan, and the differences were statistically significant,* t=-6.214, -3.055, p=0.001, 0.014*. The V_15_, V_20_, V_25_, V_30_, V_35_, and V_50_ of B-P in the IMRT plan were higher than the ARC plan, and the differences were statistically significant,* t=5.053, 8.629, 4.816, 3.998, 2.804, 3.748, p=0.001, 0.001, 0.001, 0.003, 0.021, 0.005*. The other dosimetric parameters of B-P in the IMRT plan had no statistical differences compared with the ARC plan*; *see Table 6 for details.

### 4.6. IMRT Plan versus CRT+IMRT Plan

As shown in Table 7, all the dosimetric parameters of PTV in the IMRT plan had no statistical differences compared with the CRT+IMRT plan.

As for OARs, the maximum dose of spinal cord in the CRT plan was lower than IMRT plan, and the difference was statistically significant,* t=-3.257, p=0.010*. The maximum dose and the V_50_ (%) of esophagus in the IMRT plan had no statistical differences compared with the CRT+IMRT plan. The V_40_ (%) of heart in the IMRT plan was lower than the CRT+IMRT plan, and the difference was statistically significant,* t=-2.826, p=0.020*. The other dosimetric parameters of heart in the IMRT plan had no statistical differences compared with the CRT+IMRT plan. The V_5_ (%), V_10_ (%), V_13_ (%), V_20_ (%), and V_30_ (%) of normal lung in the IMRT plan were lower than the CRT+IMRT plan, and the differences were statistically significant,* t=2.325, 2.635, 4.569, 3.806, -3.661, p=0.045, 0.027, 0.001, 0.004, 0.005*. However, the V_20_ (%) of normal lung in the IMRT plan was higher than the CRT+IMRT plan, and the difference was statistically significant,* t=2.529, p=0.032.* The V_13_ (%), V_30_ (%), and the mean dose of normal lung in the IMRT plan had no statistical differences compared with the CRT+IMRT plan, see Table 7 for details.

As for B-P, the V_5_ and V_10_ of B-P in the IMRT plan were higher than the CRT+IMRT plan, and the differences were statistically significant,* t=4.936, 5.371, p=0.001, 0.001*. The V_25_, V_30_, V_35_, V_40_, V_45_, and V_50_ of B-P in the IMRT plan were lower than IMRT plan, and the differences were statistically significant,* t=-3.705, -4.924, -3.716, -2.322, -1.857, -2.369, p=0.005, 0.001, 0.005, 0.045, 0.046, 0.042*. The other dosimetric parameters of B-P in the IMRT plan had no statistical differences compared with the CRT+IMRT plan*; *see Table 7 for details.

### 4.7. IMRT Plan versus CRT+ARC Plan

As shown in Table 8, the minimum dose, the Conformity index, and the Homogeneity index of PTV in the IMRT pan were worse than the CRT+ARC plan, and the differences were statistically significant,* t=-6.509, -4.763, 6.611, p=0.001, 0.001, 0.001. *The other dosimetric parameters of PTV in the IMRT plan had no statistical differences compared with the CRT+ARC plan.

As for OARs, the maximum dose of spinal cord, the maximum dose, and the V_50_ (%) of esophagus in the IMRT plan had no statistical differences compared with the CRT+ARC plan. The V_30_ (%) of heart in the IMRT plan was lower than the CRT+ARC plan, and the difference was statistically significant,* t=-1.936, p=0.006*. The other dosimetric parameters of heart in the IMRT plan had no statistical differences compared with the CRT+ARC plan. The V_10_ (%), V_13_ (%), V_20_ (%), V_30_ (%), and mean dose of normal lung in the IMRT plan were higher than the CRT+ARC plan, and the differences were statistically significant,* t=2.635, 4.569, 3.806, -3.661, 2.153, p=0.001, 0.001, 0.001, 0.006, 0.003*. But the V_5_ of normal lung in the IMRT plan had no statistical differences compared with the CRT+ARC plan*; *see Table 8 for details.

As for B-P, the V_15_, V_20_, V_25_, V_30_, V_35_, and V_50_ of B-P in the IMRT plan were higher than the CRT+ARC plan, and the differences were statistically significant,* t=0.616, -0.794, -3.705, -4.924,*-3.716*, p=0.042, 0.025, 0.001, 0.001, 0.005*. The other dosimetric parameters of B-P in the IMRT plan had no statistical differences compared with the CRT+ARC plan*; *see Table 8 for details.

### 4.8. ARC Plan versus CRT+IMRT Plan

As shown in Table 9, the minimum dose, the median dose, the conformity index, and the homogeneity index of PTV in the ARC plan were better than the CRT+IMRT plan, and the differences were statistically significant,* t=3.861, 2.480, 8.381, -2.795, p=0.004, 0.035, 0.001, 0.021. *The other dosimetric parameters of PTV in the ARC plan had no statistical differences compared with the CRT+IMRT plan.

As for OARs, the maximum dose of spinal cord, the maximum dose and the V_50_ (%) of esophagus in the ARC plan had no statistical differences compared with the CRT+IMRT plan. The V_20_ (%) and the median dose of heart in the ARC plan were lower than the CRT+IMRT plan, and the differences were statistically significant,* t=-2.591, -2.651, p=0.029, 0.026*. The other dosimetric parameters of heart in the ARC plan had no statistical differences compared with the CRT+IMRT plan. The V_5_ (%), V_10_ (%), V_13_ (%), and V_20_ (%) of normal lung in the ARC plan were higher than the CRT+IMRT plan, and the differences were statistically significant,* t=11.829, 3.791, 4.954, 3.185, -3.761, p=0.001, 0.004, 0.001, 0.011, 0.004*. But the V_30_ (%) in the ARC plan was lower than the CRT+IMRT plan, and the difference was statistically significant,* t=-3.761, p=0.004*. The mean dose of normal lung in the ARC plan had no statistical differences compared with the CRT+IMRT plan*; *see Table 9 for details.

As for B-P, the V_5_ and V_10_ of B-P in the ARC plan were higher than the CRT+IMRT plan, and the differences were statistically significant,* t=10.143, 6.824, p=0.001, 0.001*. However, the V_20_, V_25_, V_30_, V_35_,V_40_, V_45_, and V_50_ of B-P in the ARC plan were lower than the CRT+IMRT plan, and the differences were statistically significant,* t=-4.684, -6.648, -5.754, -3.921, -2.397, -2.136, 10.143, p=0.001, 0.001, 0.001, 0.004, 0.040, 0.049, 0.007*. The V_15_ of B-P in the ARC plan had no statistical differences compared with the CRT+IMRT plan*; *see Table 9 for details.

### 4.9. ARC Plan versus CRT+ARC Plan

As shown in Table 10, the conformity index of PTV in the ARC pan was better than the CRT+ARC plan, and the difference was statistically significant,* t=3.500, p=0.007. *The other dosimetric parameters of PTV in the ARC plan had no statistical differences compared with the CRT+ARC plan.

As for OARs, the maximum dose of spinal cord in the ARC plan was lower than the CRT+ARC plan, and the difference was statistically significant,* t=-6.857, p=0.001*. The maximum dose and the V_50_ (%) of esophagus in the ARC plan had no statistical differences compared with the CRT+ARC plan. The V_20_ (%) and the V_30_ (%) of heart in the ARC plan were lower than the CRT+ARC plan, and the differences were statistically significant,* t=-2.592, -3.080, p=0.029, 0.013*. The other dosimetric parameters of heart in the ARC plan had no statistical differences compared with the CRT+ARC plan. The V_5_ (%), V_10_ (%), V_13_ (%), and V_20_ (%) of normal lung in the ARC plan were higher than the CRT+ARC plan, and the differences were statistically significant,* t=4.346, 8.832, 5.856, 6.281, p=0.002, 0.001, 0.001, 0.001*. But the V_30_ (%) in the ARC plan was lower than the CRT+ARC plan, and the difference was statistically significant,* t=-4.461, p=0.002*. The mean dose of normal lung in CRT plan had no statistical difference compared with the IMRT plan*; *see Table 10 for details.

As for B-P, the V_25_, V_30_, and V_35_ of B-P in the ARC plan were lower than the CRT+ARC plan, and the differences were statistically significant,* t=-6.512,-6.645, -3.973, p=0.001, 0.001, 0.003*. The other dosimetric parameters of B-P in the ARC plan had no statistical differences compared with the CRT+ARC plan*; *see Table 10 for details.

### 4.10. CRT+IMRT Plan versus CRT+ARC Plan

As shown in Table 11, the minimum dose, the conformity index, and the homogeneity index of PTV in the CRT+IMRT plan were worse than the CRT+ARC plan, and the differences were statistically significant,* t=-6.053, -7.334, 5.364, p=0.001, 0.001, 0.001. *The other dosimetric parameters of PTV in the CRT+IMRT plan had no statistical differences compared with the CRT+ARC plan.

As for OARs, the maximum dose of spinal cord in the CRT+IMRT plan was higher than the CRT+ARC plan, and the difference was statistically significant,* t=3.744, p=0.005*. The dosimetric parameters of the esophagus and the heart in the CRT+IMRT plan had no statistical differences compared with the CRT+ARC plan. The V_10_ (%), V_13_ (%), V_20_ (%), and the mean dose of normal lung the CRT+IMRT plan were higher than the CRT+ARC plan, and the differences were statistically significant,* t=3.546, 3.376, 7.026, p=0.006, 0.008, 0.001*. The V_30_ (%) and the mean dose of normal lung the CRT+IMRT plan had no statistical differences compared with the CRT+ARC plan*; *see Table 11 for details.

As for B-P, all the dosimetric parameters of B-P in the CRT+IMRT plan had no statistical differences compared with the CRT+ARC plan*; *see Table 11 for details.

## 5. Discussion

As shown in the above results, the conformity index of PTV in the CRT plan was worse than all the other plans, the IMRT, ARC, CRT+IMRT, and CRT+ARC plan. By the CRT plan compared with the IMRT and CRT+IMRT plan, the minimum dose of PTV was better. Nevertheless, as for PTV, the CRT plan compared with the ARC and CRT+ARC plan had no any dosimetry advantages. As for OARs, the CRT plan had dosimetry advantages in esophagus than all the other plans. The CRT plan had dosimetry advantages in the V_30_ (%) of heart than the CRT+ARC plan. But as for the other OARs, the CRT plan had no any dosimetry advantages compared with all the other plans. As for B-P, the dosimetry parameters in the CRT plan were worse than the IMRT and ARC plan, but had no statistical differences compared with the CRT+IMRT and the CRT+ARC plan.

The studies [5–8] about PORT in stage IIIA-N2 NSCLC patients in recent years mostly adopted the CRT technique. Though the CRT is relatively safe and stable with higher marginal dose of tumor, the dosimetry advantages of the CRT are not obvious as discussed above, perhaps due to the limitations of the technique itself. In addition, if the volume of PTV is a little larger, it maybe not meet the requirement of prescription dose and make the organ not exceeding the limit dose at the same time.

As the IMRT plan compared with all the other plans, the minimum dose of PTV in the IMRT plan was worse than the CRT plan, but the conformity index was better. The dosimetry parameters of PTV in the IMRT plan had no statistical differences compared with the CRT+IMRT plan. The minimum dose, the conformity index, and the homogeneity index of PTV in the IMRT plan were worse than the ARC plan and the CRT+ARC plan. As for OARs, except the maximum dose of esophagus, the dose of all the other OARs in the IMRT plan was lower than the CRT plan. The dose of spinal cord and heart in the IMRT plan was lower than the CRT+IMRT plan, but the dose of esophagus and normal lung in the IMRT plan was higher than the CRT+IMRT plan. The dosimetry parameters of spinal cord and esophagus in the IMRT plan had no statistical differences compared with the ARC and the CRT+ARC plan. The V_20_ (%) and V_30_ (%) of heart in the IMRT plan were higher than the ARC plan. The V_20_ (%) of normal lung in the IMRT plan was higher than the ARC plan, but the V_5_ (%) and V_10_ (%) were lower than the ARC plan. The V_30_ (%) of heart in the IMRT plan was higher than the CRT+ARC plan, but the V_10_ (%), V_15_ (%), V_20_ (%), V_30_ (%), and mean dose of normal lung in the IMRT plan were all higher than the CRT+ARC plan. As for B-P, the dosimetry parameters in the IMRT plan were all lower than the CRT plan. The V_20~50_ of B-P in the IMRT plan were all lower than the CRT+ IMRT plan, but the V_5~10_ were higher than the CRT+ IMRT plan. The V_15~35_ of B-P in the IMRT plan were all higher than the ARC plan, but the V_5~10_ were lower than the ARC plan. The V_20~35_ of B-P in the IMRT plan were all lower than the ARC plan, but the V_15_ was higher than the ARC plan.

The studies about PORT in stage IIIA-N2 NSCLC patients in recent years which adopted the IMRT technique were not many. Abigail T Berman et al. [9] reported an in silico comparative analysis of passive scattering proton therapy (PSPT) and intensity modulated proton therapy (IMPT) with intensity modulated photon beam radiotherapy (IMRT) PORT and concluded that IMPT demonstrates a large decrease in dose to all OARs, while PSPT reduces the low dose lung bath and increases the volume of lung receiving high dose, and reductions are seen in dosimetric parameters predictive of radiation pneumonitis and cardiac morbidity and mortality, and this reduction may correlate with a decrease in dose-limiting toxicity and improve the therapeutic ratio. Jill S. Remick et al. [10] investigated the survival outcomes and early toxicity profile of PORT with proton beam therapy (PBT) versus IMRT for non-small-cell lung cancer in a cohort of 61 patients with positive microscopic margins and/or positive N2 lymph nodes and found that postoperative PBT in locally advanced NSCLC is well-tolerated and has similar excellent short-term outcomes when compared with IMRT. In the above two studies, IMRT had no dosimetry advantages compared with IMPT, PSPT, and PBT. In our study as discussed above, the IMRT had certain dosimetry advantages compared with CRT, CRT+IMRT, and CRT+ARC.

Comparing the ARC plan with all other plans, the ARC plan had dosimetry advantages in PTV than all the other plans. As for OARs, the ARC plan had dosimetry advantages in spinal cord, heart and normal lung compared with the CRT plan, but had no dosimetry advantages in esophagus. Nevertheless, when compared with the CRT+ARC plan, the ARC plan had dosimetry advantages in spinal cord, heart and V_30_ (%) of normal lung, and had no dosimetry advantages in V_5_ (%), V_10_ (%), V_13_ (%), and V_20_ (%) of normal lung. The ARC plan had dosimetry advantages in heart and V_20_ (%) of normal lung compared with the IMRT plan, but had no dosimetry advantages in V_5_ (%) and V_10_ (%) of normal lung. As well as compared with the CRT+IMRT plan, the ARC plan had dosimetry advantages in spinal cord, heart, and V_30_ (%) of normal lung and had no dosimetry advantages in V_5_ (%),V_10_ (%), V_13_ (%), and V_20_ (%) of normal lung. As for B-P, the ARC plan had dosimetry advantages compared to the CRT plan and the CRT+ARC plan. The ARC plan had no dosimetry advantages in V_5_ (%) and V_10_ (%) of B-P compared with the IMRT plan and the CRT+IMRT plan, but had dosimetry advantages in V_15~35_ (%) of B-P compared to the IMRT plan, and had dosimetry advantages in V_20~50_ (%) of B-P compared to the CRT+IMRT plan.

The studies about PORT in stage IIIA-N2 NSCLC patients in recent years that adopted the ARC technique were fewer. Huan-Huan Wang et al. [11] had evaluated the ideal timing of PORT in the management of completely resected (R0) Stage IIIA-N2 NSCLC. In their study, PORT was administered using not only CRT and IMRT, but also VMAT (ARC). Nevertheless, the study did not give detailed technique parameters and patients were not grouped according to different techniques. In our study as discussed above, the ARC plan had absolute dosimetry advantages in PTV, but had no dosimetry advantages in low dose area of normal lung and B-P.

Comparing the CRT+IMRT plan with all other plans, the CRT+IMRT plan had no dosimetry differences in PTV compared to the IMRT plan and had no dosimetry advantages in PTV compared with the ARC and CRT+ARC plan. The median dose and conformity of PTV in the CRT+IMRT were better than the CRT plan, but the minimum dose and homogeneity index PTV in the CRT+IMRT were worse than the CRT plan. As for OARs, the CRT+IMRT plan had no dosimetry advantages in esophagus compared to the CRT plan, but had dosimetry advantages in heart and normal lung compared with the CRT plan. The CRT+IMRT plan compared with the IMRT plan and ARC plan had no dosimetry advantages in spinal cord, heart, and the V_30_ (%) of normal lung, but had dosimetry advantages in the V_5_ (%), V_10_ (%), V_13_ (%), and V_20_ (%) of normal lung. The CRT+IMRT plan had no dosimetry advantages in all the OARs compared with the CRT +ARC plan. As for B-P, the CRT+IMRT plan had dosimetry advantages except the V_30_ (%) of B-P compared to the CRT plan. As for the CRT+IMRT plan compared with the IMRT and ARC plan, the CRT+IMRT plan had dosimetry advantages in low dose area of B-P and had no dosimetry advantages in high dose area of B-P. The CRT+IMRT plan had no dosimetry differences compared to the CRT +ARC plan.

There was no study about PORT in stage IIIA-N2 NSCLC patients in recent years which adopted the CRT+IMRT technique. Gerrit J. Blom et al. [12] compared the dosimetry characteristics of Hybrid-IMRT(CRT+IMRT) with RapidArc (ARC) in locally advanced non-small-cell lung cancer. It was challenging for 60-66Gy to deliver to large target volumes due to the need to spare OARs, so Hybrid-IMRT is currently their standard technique in locally advanced non-small-cell lung cancer. Though the target volumes in PORT patients were not very large and the prescriptions were not very high generally, the dose limits of OARs were lower than patients who had no surgery. Therefore, investigating the dosimetry characteristics of the CRT+IMRT technique in PORT patients is also necessary.

The CRT+ARC plan had dosimetry advantages in PTV compared to the CRT plan, IMRT plan, and CRT+IMRT plan, but the conformal index of PTV in the CRT+ARC plan was worse than the ARC plan. As for OARs, the CRT+ARC plan had no dosimetry advantages in spinal cord and the V_30_ (%) of heart compared with the CRT plan, but had dosimetry advantages in V_20_ (%), V_40_ (%), V_45_ (%), and mean dose of heart and normal lung. The CRT+ARC plan had dosimetry advantages in normal lung compared with the IMRT plan. CRT+ARC plan had no dosimetry advantages in spinal cord and the V_30_ (%) of normal lung compared with the ARC plan, but had dosimetry advantages in heart and V_5_ (%), V_10_ (%), V_13_ (%), and V_20_ (%) of normal lung. The CRT+ARC plan had dosimetry advantages in spinal cord and normal lung compared to the CRT+IMRT plan. As for B-P, the CRT+ARC plan had dosimetry advantages in V_25~35_ of B-P compared to the CRT plan, and had dosimetry advantages in V_10~15_ of B-P compared to the IMRT plan. The CRT+ARC plan had no dosimetry advantages in B-P compared with the ARC plan, and had no dosimetry differences in B-P compared with the IMRT plan.

There was also no study about PORT in stage IIIA-N2 NSCLC patients in recent years which adopted the CRT+ARC technique. John Agapito [13] compared the dosimetry characteristics of CRT with hybrid-VMAT (CRT+ARC) in locally advanced non-small-cell lung cancer, and concluded that the hybrid technique shows promise, but the quantity assurance implications of motion at treatment needs careful consideration. As discussed above, the CRT+ARC plan compared with the ARC plan, though conformal index of PTV was worse, the low dose area of normal lung was better. The studies by Len AM. et al. [14] and Schallen kamp JM. et al. [15] indicated that the radiation pneumonitis was related to the low dose areas of normal lung. If the low dose areas of ARC plan for a patient exceed the limits dose, a CRT+ARC plan may be a better choice.

## 6. Conclusion

According to the above analysis, no plan had absolute dosimetry advantages than any other plans. In the clinic, the plans could be chosen according to their dosimetry characteristics. For example, for a patient who needs protection especially for esophagus, the CRT plan could be chosen. If a plan for a patient needs a good dosimetry distribution for PTV, the ARC plan could be chosen. Another example is that the volume of PTV is a little large and the lung of a patient needs better protection, then the CRT+ARC plan could be chosen. Of course, in the course of implementation of the above radiotherapy techniques, if modern auxiliary equipment can be incorporated with them, they can be better implemented in patients. The modern auxiliary equipment includes respiratory gate control [16, 17], CBCT image guidance technology [18–20], and so on. In future clinical studies, we hope to see more applications of these advanced radiotherapy techniques and radiotherapy auxiliary equipment. Moreover, detailed technical parameters can be given, and each radiotherapy technique can be grouped differently to study.

## Figures and Tables

**Table 1:** General clinical data of patients.

Case no.	Age (yeas)	Sex	T stage	N stage	M stage	Clinical stage	Pathological pattern	PTV volume (cc)
01	Female	68	2	2	0	IIIA	Adenocarcinoma	220.5
02	Female	63	2	2	0	IIIA	Adenocarcinoma	197.0
03	Male	64	3	2	0	IIIA	Adenocarcinoma	373.3
04	Male	65	3	2	0	IIIA	Squamous cell carcinoma	430.8
05	Male	65	3	2	0	IIIA	Squamous cell carcinoma	389.1
06	Male	69	2	2	0	IIIA	Squamous cell carcinoma	293.9
07	Male	51	2	2	0	IIIA	Squamous cell carcinoma	184.7
08	Female	63	1b	2	0	IIIA	Adenocarcinoma	178.2
09	Male	67	3	2	0	IIIA	Large cell carcinoma	264.0
10	Male	62	1a	2	0	IIIA	Adenocarcinoma	367.7

**Table 2:** Comparisons of PTV, OAR, and B-P dosimetric parameters between CRT plan and IMRT plan (cGy, $\overset{-}{x}\pm s$).

Dosimetric parameters	CRT plan	IMRT plan	Difference	*t* value	*P* value
PTV					
Minimum dose/cGy	4353.71±69.41	3936.34±189.77	417.37±197.05	6.698	0.001
Maximum dose/cGy	5397.29 ±82.69	5350.08±61.15	47.21±108.72	1.373	0.203
Median dose/cGy)	5001.56±14.33	4979.08±77.62	22.48±81.23	0.875	0.404
Conformity index	0.69±0.05	0.80±0.04	-0.11±0.06	-5.367	0.001
Homogeneity index	0.21±0.02	0.28±0.04	-0.00±0.03	-0.330	0.749
V_105_ (%)	15.11±3.64	2.92±4.38	12.19±16.34	2.359	0.043
OAR					
Spinal cord Maximum dose	3214.23±285.34	2636.20±179.98	578.02±259.87	7.034	0.001
Esophagus					
V_50_ (%)	8.49±9.94	17.79±14.08	-9.30±16.53	-1.778	0.109
Maximum dose	5110.67±113.32	5270.17±109.33	-159.50±132.28	-3.813	0.004
Heart					
V_20_ (%)	23.77±21.93	20.78±21.65	2.97±1.75	5.382	0.001
V_30_ (%)	11.98±12.97	9.83±10.15	2.15±3.10	2.191	0.056
V_40_ (%)	6.92±7.43	4.87±5.24	2.05±2.26	2.864	0.019
V_45_ (%)	5.15±5.48	3.59±3.84	1.55±1.73	2.832	0.020
Mean dose	1061.75±777.82	932.07±702.92	129.68±83.46	4.913	0.001
Normal lung					
V_5_ (%)	54.24±8.06	49.59±6.17	4.64±2.27	6.456	0.001
V_10_ (%)	37.28±8.74	35.13±7.42	2.14±2.55	2.653	0.026
V_13_ (%)	33.62±7.95	30.31±6.38	3.29±2.25	4.636	0.001
V_20_ (%)	26.62±7.23	22.22±4.48	4.40±2.32	5.982	0.001
V_30_ (%)	11.59±1.64	8.63±1.47	2.95±1.38	6.771	0.001
Mean dose	1006.29±522.53	1040.41±147.82	-34.12±418.64	-0.258	0.802
B-P					
V_5_(%)	27.31±8.42	22.91±2.48	4.39±7.40	1.879	0.093
V_10_(%)	18.97±5.53	15.73±2.21	3.24±4.37	2.347	0.044
V_15_(%)	16.03±4.77	12.55±1.83	3.48±3.84	2.870	0.018
V_20_(%)	12.49±4.87	9.29±1.79	3.19±4.28	2.363	0.042
V_25_(%)	7.33±3.41	5.19±1.40	2.13±3.05	2.216	0.054
V_30_(%)	4.29±2.76	2.73±0.81	1.56±2.45	2.015	0.075
V_35_(%)	2.94±2.29	1.51±0.46	1.44±1.97	2.317	0.046
V_40_(%)	1.95±1.32	0.71±0.20	1.24±1.23	3.181	0.011
V_45_(%)	1.24±1.06	0.28±0.10	0.95±1.01	2.957	0.016
V_50_(%)	0.23±0.24	0.03±0.03	0.19±.22	2.771	0.022

**Table 3:** Comparisons of PTV, OAR, and B-P dosimetric parameters between CRT plan and ARC plan $\left(cGy,\;\overset{-}{x}\pm s\right)$.

Dosimetric parameters	CRT plan	ARC plan	Difference	*t *value	*P *value
PTV					
Minimum dose/cGy	4353.71±69.41	4285.12±159.09	68.59±165.02	1.314	0.221
Maximum dose/cGy	5397.29 ±82.69	5348.30±61.22	48.99±100.59	1.540	0.158
Median dose/cGy)	5001.56±14.33	5025.60±6.27	-24.04±17.14	-4.433	0.002
Conformity index	0.69±0.05	0.88±0.02	-0.19±0.04	-15.148	0.001
Homogeneity index	0.21±0.02	0.21±0.04	-0.00±0.04	-0.330	0.749
V_105_ (%)	15.11±3.64	0.74±.84	14.37±16.43	2.766	0.022
OAR					
Spinal cord Maximum dose	3214.23±285.34	2383.08±353.89	831.15±476.47	5.516	0.001
Esophagus					
V_50_ (%)	8.49±9.94	19.48±12.51	-10.99±8.11	-4.286	0.002
Maximum dose	5110.67±113.32	4863.6±1253.24	-611.56±1105.668	0.651	0.531
Heart					
V_20_ (%)	23.77±21.93	13.98±13.25	3.21±16.34	3.369	0.008
V_30_ (%)	11.98±12.97	6.66±6.03	0.29±10.33	2.393	0.040
V_40_ (%)	6.92±7.43	3.79±3.21	0.04±6.23	2.295	0.047
V_45_ (%)	5.15±5.48	2.89±2.45	0.01±4.49	2.278	0.049
Mean dose	1061.75±777.82	698.58±652.93	100.58±625.75	3.129	0.012
Normal lung					
V_5_ (%)	54.24±8.06	56.06±6.17	-5.33±1.66	-1.186	0.266
V_10_ (%)	37.28±8.74	36.61±7.24	-1.71±3.07	0.643	0.536
V_13_ (%)	33.62±7.95	29.20±6.20	1.82±7.01	3.860	0.004
V_20_ (%)	26.62±7.23	19.49±3.34	3.59±10.68	4.551	0.001
V_30_ (%)	11.59±1.64	8.85±0.90	1.55±3.91	5.244	0.001
Mean dose	1006.29±522.53	972.39±362.74	-344.47±412.264	0.203	0.844
B-P					
V_5_(%)	27.31±8.42	26.15±3.14	-3.97±6.30	0.514	0.619
V_10_(%)	18.97±5.53	16.54±2.41	-0.86±5.72	1.667	0.130
V_15_(%)	16.03±4.77	10.97±1.80	2.28±7.82	4.121	0.003
V_20_(%)	12.49±4.87	6.46±1.43	3.22±8.84	4.861	0.001
V_25_(%)	7.33±3.41	3.72±0.93	1.64±5.58	4.142	0.003
V_30_(%)	4.29±2.76	2.13±0.55	0.39±3.92	2.774	0.022
V_35_(%)	2.94±2.29	1.22±0.29	0.18±3.27	2.532	0.032
V_40_(%)	1.95±1.32	0.66±0.14	0.39±2.19	3.244	0.010
V_45_(%)	1.24±1.06	0.25±0.04	0.24±1.74	2.999	0.015
V_50_(%)	0.23±0.24	0.00±0.00	0.01±0.05	3.748	0.005

**Table 4:** Comparisons of PTV, OAR, and B-P dosimetric parameters between CRT plan and CRT+IMRT plan $\left(cGy,\;\overset{-}{x}\pm s\right)$.

Dosimetric parameters	CRT plan	CRT+IMRT plan	Difference	*t *value	*P *value
PTV					
Minimum dose/cGy	4353.71±69.41	4051.12±183.68	302.59±62.84	4.815	0.001
Maximum dose/cGy	5397.29 ±82.69	5373.66±103.43	23.63±50.75	0.466	0.653
Median dose/cGy)	5001.56±14.33	5017.78±9.25	-16.22±6.23	-2.604	0.029
Conformity index	0.69±0.05	0.80±0.03	-0.11±0.04	-7.910	0.001
Homogeneity index	0.21±0.02	0.27±0.05	-0.06±0.06	-3.006	0.015
V_105_ (%)	15.11±3.64	3.48±5.16	11.63±19.11	1.924	0.086
OAR					
Spinal cord Maximum dose	3214.23±285.34	3181.48±556.33	32.75±188.93	0.173	0.866
Esophagus					
V_50_ (%)	8.49±9.94	14.68±13.39	-6.19±10.77	-1.817	0.103
Maximum dose	5110.67±113.32	5205.15±96.40	-94.48±109.08	-2.739	0.023
Heart					
V_20_ (%)	23.77±21.93	20.76±21.46	3.00±3.26	2.913	0.017
V_30_ (%)	11.98±12.97	13.12±15.14	-1.14±2.68	-1.347	0.211
V_40_ (%)	6.92±7.43	5.32±5.49	1.60±2.11	2.391	0.041
V_45_ (%)	5.15±5.48	3.86±4.20	1.29±1.36	2.996	0.015
Mean dose	1061.75±777.82	983.30±742.64	78.45±83.12	2.985	0.015
Normal lung					
V_5_ (%)	54.24±8.06	46.88±7.19	7.35±4.28	5.435	0.001
V_10_ (%)	37.28±8.74	31.74±6.64	5.54±5.24	3.342	0.009
V_13_ (%)	33.62±7.95	24.71±4.66	8.90±5.54	5.082	0.001
V_20_ (%)	26.62±7.23	17.95±2.56	8.67±5.32	5.153	0.001
V_30_ (%)	11.59±1.64	11.34±2.11	0.24±1.68	0.457	0.659
Mean dose	1006.29±522.53	1001.52±127.12	4.77±447.35	0.034	0.974
B-P					
V_5_(%)	27.31±8.42	20.49±2.71	6.83±7.07	3.053	0.014
V_10_(%)	18.97±5.53	14.32±2.43	4.65±3.90	3.780	0.004
V_15_(%)	16.03±4.77	11.92±3.92	4.11±1.13	11.481	0.001
V_20_(%)	12.49±4.87	10.04±3.22	2.45±1.81	4.287	0.002
V_25_(%)	7.33±3.41	8.32±2.76	-0.98±1.56	-1.992	0.078
V_30_(%)	4.29±2.76	5.76±2.33	-1.47±1.16	-3.967	0.003
V_35_(%)	2.94±2.29	3.27±1.80	-0.32±0.78	-1.282	0.232
V_40_(%)	1.95±1.32	1.72±1.46	0.24±0.24	3.209	0.011
V_45_(%)	1.24±1.06	0.87±1.03	0.37±0.12	9.582	0.001
V_50_(%)	0.23±0.24	0.06±0.06	0.17±0.20	2.730	0.023

**Table 5:** Comparisons of PTV, OAR, and B-P dosimetric parameters between IMRT plan and ARC plan $\left(cGy,\overset{-}{x}\pm s\right)$.

Dosimetric parameters	CRT plan	CRT+ARC plan	Difference	*t *value	*P *value
PTV					
Minimum dose/cGy	4353.71±69.41	4305.23±114.48	48.48±127.34	1.204	0.259
Maximum dose/cGy	5397.29 ±82.69	5328.32±58.00	68.97±101.56	2.148	0.060
Median dose/cGy)	5001.56±14.33	5068.44±77.68	-66.88±73.16	-2.891	0.018
Conformity index	0.69±0.05	0.86±0.02	-0.18±0.05	-12.227	0.001
Homogeneity index	0.21±0.02	0.20±0.03	0.00±0.04	0.260	0.801
V_105_ (%)	15.11±3.64	3.75±9.42	11.36±19.94	1.801	0.105
OAR					
Spinal cord Maximum dose	3214.23±285.34	2870.81±535.92	343.42±622.22	1.745	0.115
Esophagus					
V_50_ (%)	8.49±9.94	17.02±12.75	-8.53±9.27	-2.911	0.017
Maximum dose	5110.67±113.32	5229.34±79.93	-118.67±125.23	-2.997	0.015
Heart					
V_20_ (%)	23.77±21.93	20.56±21.20	3.20±3.45	2.933	0.017
V_30_ (%)	11.98±12.97	14.39±13.89	-2.41±2.16	-3.529	0.006
V_40_ (%)	6.92±7.43	4.88±4.73	2.05±2.80	2.310	0.046
V_45_ (%)	5.15±5.48	2.94±2.42	2.21±3.18	2.196	0.056
Mean dose	1061.75±777.82	872.64±788.90	189.11±256.43	2.332	0.045
Normal lung					
V_5_ (%)	54.24±8.06	48.77±9.22	5.47±5.55	3.114	0.012
V_10_ (%)	37.28±8.74	28.30±5.43	8.98±4.48	6.330	0.001
V_13_ (%)	33.62±7.95	22.40±4.28	11.22±5.32	6.673	0.001
V_20_ (%)	26.62±7.23	14.74±2.08	11.89±5.94	6.319	0.001
V_30_ (%)	11.59±1.64	10.36±0.81	1.23±1.56	2.494	0.034
Mean dose	1006.29±522.53	952.15±120.92	54.14±452.68	0.378	0.714
B-P					
V_5_(%)	27.31±8.42	25.22±12.40	2.10±14.42	0.460	0.656
V_10_(%)	18.97±5.53	14.86±6.45	4.11±7.20	1.806	0.104
V_15_(%)	16.03±4.77	10.13±4.22	5.91±5.02	3.719	0.005
V_20_(%)	12.49±4.87	7.99±2.65	4.50±4.37	3.261	0.010
V_25_(%)	7.33±3.41	6.83±1.99	0.50±2.90	0.546	0.598
V_30_(%)	4.29±2.76	5.33±1.80	-1.03±3.03	-1.077	0.309
V_35_(%)	2.94±2.29	3.42±1.89	-0.47±2.97	-0.501	0.628
V_40_(%)	1.95±1.32	1.58±1.71	0.37±2.10	0.560	0.589
V_45_(%)	1.24±1.06	0.68±1.19	0.57±1.55	1.149	0.280
V_50_(%)	0.23±0.24	0.12±0.33	0.12±0.39	0.933	0.375

**Table 6:** Comparisons of PTV, OAR, and B-P dosimetric parameters between IMRT plan and ARC plan $\left(cGy,\;\overset{-}{x}\pm s\right)$.

Dosimetric parameters	IMRT plan	ARC plan	Difference	*t *value	*P *value
PTV					
Minimum dose/cGy	3936.34±189.77	4285.12±159.09	-348.78±200.06	-5.513	0.001
Maximum dose/cGy	5350.08±61.15	5348.30±61.22	1.78±50.53	0.111	0.914
Median dose/cGy)	4979.08±77.62	5025.60±6.27	-46.52±78.50	-1.874	0.094
Conformity index	0.80±0.04	0.88±0.02	-0.08±0.05	-5.381	0.001
Homogeneity index	0.28±0.04	0.21±0.04	0.07±0.05	4.768	0.001
V_105_ (%)	2.92±4.38	0.74±.84	2.18±3.80	1.812	0.103
OAR					
Spinal cord Maximum dose	2636.20±179.98	2383.08±353.89	253.12±378.72	2.114	0.064
Esophagus					
V_50_ (%)	17.79±14.08	19.48±12.51	-1.69±11.39	-0.469	0.650
Maximum dose	5270.17±109.33	4863.6±1253.24	406.55±1182.83	1.087	0.305
Heart					
V_20_ (%)	20.78±21.65	13.98±13.25	6.80±8.88	2.423	0.038
V_30_ (%)	9.83±10.15	6.66±6.03	3.16±4.18	2.391	0.040
V_40_ (%)	4.87±5.24	3.79±3.21	1.09±2.11	1.628	0.138
V_45_ (%)	3.59±3.84	2.89±2.45	0.70±1.44	1.530	0.160
Mean dose	932.07±702.92	698.58±652.93	233.48±338.59	2.181	0.057
Normal lung					
V_5_ (%)	49.59±6.17	56.06±6.17	-6.47±3.86	-5.309	0.001
V_10_ (%)	35.13±7.42	36.61±7.24	-1.47±1.72	-2.697	0.025
V_13_ (%)	30.31±6.38	29.20±6.20	1.12±2.19	1.619	0.140
V_20_ (%)	22.22±4.48	19.49±3.34	2.73±3.41	2.529	0.032
V_30_ (%)	8.63±1.47	8.85±0.90	-0.22±1.52	-0.467	0.651
Mean dose	1040.41±147.82	972.39±362.74	68.01±283.07	0.760	0.467
B-P					
V_5_(%)	22.91±2.48	26.15±3.14	-3.22±1.64	-6.214	0.001
V_10_(%)	15.73±2.21	16.54±2.41	-0.81±0.83	-3.055	0.014
V_15_(%)	12.55±1.83	10.97±1.80	1.57±0.98	5.053	0.001
V_20_(%)	9.29±1.79	6.46±1.43	2.84±1.04	8.629	0.001
V_25_(%)	5.19±1.40	3.72±0.93	1.48±0.97	4.816	0.001
V_30_(%)	2.73±0.81	2.13±0.55	0.60±0.47	3.998	0.003
V_35_(%)	1.51±0.46	1.22±0.29	0.28±0.32	2.804	0.021
V_40_(%)	0.71±0.20	0.66±0.14	0.05±0.14	1.172	0.271
V_45_(%)	0.28±0.10	0.25±0.04	0.04±0.09	1.404	0.194
V_50_(%)	0.03±0.03	0.00±0.00	0.03±0.02	3.748	0.005

**Table 7:** Comparisons of PTV, OAR, and B-P dosimetric parameters between IMRT plan and CRT+IMRT plan $\left(cGy,\overset{-}{x}\pm s\right)$.

Dosimetric parameters	IMRT plan	CRT+IMRT plan	Difference	*t *value	*P *value
PTV					
Minimum dose/cGy	3936.34±189.77	4051.12±183.68	-114.78±178.63	-2.032	0.073
Maximum dose/cGy	5350.08±61.15	5373.66±103.43	-23.58±112.33	-0.664	0.523
Median dose/cGy)	4979.08±77.62	5017.78±9.25	-38.70±79.38	-1.542	0.158
Conformity index	0.80±0.04	0.80±0.03	0.00±0.05	0.060	0.954
Homogeneity index	0.28±0.04	0.27±0.05	0.01±0.05	1.127	0.289
V_105_ (%)	2.92±4.38	3.48±5.16	-0.56±6.53	-0.271	0.792
OAR					
Spinal cord Maximum dose	2636.20±179.98	3181.48±556.33	-545.27±529.43	-3.257	0.010
Esophagus					
V_50_ (%)	17.79±14.08	14.68±13.39	3.11±11.78	0.835	0.425
Maximum dose	5270.17±109.33	5205.15±96.40	65.02±104.19	1.973	0.080
Heart					
V_20_ (%)	20.78±21.65	20.76±21.46	0.02±2.75	0.029	0.978
V_30_ (%)	9.83±10.15	13.12±15.14	-3.29±5.38	-1.936	0.085
V_40_ (%)	4.87±5.24	5.32±5.49	-0.45±0.51	-2.826	0.020
V_45_ (%)	3.59±3.84	3.86±4.20	-0.26±0.42	-1.993	0.077
Mean dose	932.07±702.92	983.30±742.64	-51.23±83.99	-1.929	0.086
Normal lung					
V_5_ (%)	49.59±6.17	46.88±7.19	2.71±3.68	2.325	0.045
V_10_ (%)	35.13±7.42	31.74±6.64	3.39±4.07	2.635	0.027
V_13_ (%)	30.31±6.38	24.71±4.66	5.61±3.88	4.569	0.001
V_20_ (%)	22.22±4.48	17.95±2.56	4.27±3.55	3.806	0.004
V_30_ (%)	8.63±1.47	11.34±2.11	-2.71±2.34	-3.661	0.005
Mean dose	1040.41±147.82	1001.52±127.12	38.89±57.13	2.153	0.060
B-P					
V_5_(%)	22.91±2.48	20.49±2.71	2.43±1.56	4.936	0.001
V_10_(%)	15.73±2.21	14.32±2.43	1.42±0.83	5.371	0.001
V_15_(%)	12.55±1.83	11.92±3.92	0.63±3.23	0.616	0.553
V_20_(%)	9.29±1.79	10.04±3.22	-0.74±2.96	-0.794	0.448
V_25_(%)	5.19±1.40	8.32±2.76	-3.12±2.66	-3.705	0.005
V_30_(%)	2.73±0.81	5.76±2.33	-3.03±1.94	-4.924	0.001
V_35_(%)	1.51±0.46	3.27±1.80	-1.76±1.50	-3.716	0.005
V_40_(%)	0.71±0.20	1.72±1.46	-1.00±1.36	-2.322	0.045
V_45_(%)	0.28±0.10	0.87±1.03	-0.58±0.99	-1.857	0.046
V_50_(%)	0.03±0.03	0.06±0.06	0.03±0.03	-2.369	0.042

**Table 8:** Comparisons of PTV, OAR, and B-P dosimetric parameters between IMRT plan and CRT+ARC plan $\left(cGy,\overset{-}{x}\pm s\right)$.

Dosimetric parameters	IMRT plan	CRT+ARC plan	Difference	*t *value	*P *value
PTV					
Minimum dose/cGy	3936.34±189.77	4305.23±114.48	-368.89±179.22	-6.509	0.001
Maximum dose/cGy	5350.08±61.15	5328.32±58.00	21.76±62.08	1.108	0.296
Median dose/cGy)	4979.08±77.62	5068.44±77.68	-89.36±127.30	-2.220	0.054
Conformity index	0.80±0.04	0.86±0.02	-0.07±0.05	-4.763	0.001
Homogeneity index	0.28±0.04	0.20±0.03	0.08±0.04	6.611	0.001
V_105_ (%)	2.92±4.38	3.75±9.42	0.83±11.02	-0.238	0.817
OAR					
Spinal cord Maximum dose	2636.20±179.98	2870.81±535.92	-234.60±564.83	-3.257	0.222
Esophagus					
V_50_ (%)	17.79±14.08	17.02±12.75	0.76±11.38	0.835	0.836
Maximum dose	5270.17±109.33	5229.34±79.93	40.83±113.85	1.973	0.286
Heart					
V_20_ (%)	20.78±21.65	20.56±21.20	0.22±2.86	0.029	0.810
V_30_ (%)	9.83±10.15	14.39±13.89	-4.56±3.99	-1.936	0.006
V_40_ (%)	4.87±5.24	4.88±4.73	-0.01±0.75	-2.826	0.967
V_45_ (%)	3.59±3.84	2.94±2.42	0.65±1.51	-1.993	0.205
Mean dose	932.07±702.92	872.64±788.90	59.43±263.06	-1.929	0.493
Normal lung					
V_5_ (%)	49.59±6.17	48.77±9.22	0.83±5.61	2.325	0.652
V_10_ (%)	35.13±7.42	28.30±5.43	6.84±3.23	2.635	0.001
V_13_ (%)	30.31±6.38	22.40±4.28	7.92±4.03	4.569	0.001
V_20_ (%)	22.22±4.48	14.74±2.08	7.48±4.17	3.806	0.001
V_30_ (%)	8.63±1.47	10.36±0.81	-1.72±1.50	-3.661	0.006
Mean dose	1040.41±147.82	952.15±120.92	88.26±69.44	2.153	0.003
B-P					
V_5_(%)	22.91±2.48	25.22±12.40	-2.30±12.04	4.936	0.562
V_10_(%)	15.73±2.21	14.86±6.45	0.87±5.46	5.371	0.626
V_15_(%)	12.55±1.83	10.13±4.22	2.42±3.23	0.616	0.042
V_20_(%)	9.29±1.79	7.99±2.65	1.31±1.54	-0.794	0.025
V_25_(%)	5.19±1.40	6.83±1.99	-1.63±1.09	-3.705	0.001
V_30_(%)	2.73±0.81	5.33±1.80	-2.60±1.21	-4.924	0.001
V_35_(%)	1.51±0.46	3.42±1.89	-1.91±1.63	-3.716	0.005
V_40_(%)	0.71±0.20	1.58±1.71	-0.87±1.56	-2.322	0.112
V_45_(%)	0.28±0.10	0.68±1.19	-0.38±1.12	-1.857	0.302
V_50_(%)	0.03±0.03	0.12±0.33	-0.08±0.31	-2.369	0.426

**Table 9:** Comparisons of PTV, OAR, and B-P dosimetric parameters between ARC plan and CRT+IMRT plan $\left(cGy,\overset{-}{x}\pm s\right)$.

Dosimetric parameters	ARC plan	CRT+IMRT plan	Difference	*t *value	*P *value
PTV					
Minimum dose/cGy	4285.12±159.09	4051.12±183.68	234.00±191.68	3.861	0.004
Maximum dose/cGy	5348.30±61.22	5373.66±103.43	-25.36±122.01	-0.657	0.527
Median dose/cGy)	5025.60±6.27	5017.78±9.25	7.82±9.97	2.480	0.035
Conformity index	0.88±0.02	0.80±0.03	0.08±0.03	8.381	0.001
Homogeneity index	0.21±0.04	0.27±0.05	-0.05±0.06	-2.795	0.021
V_105_ (%)	0.74±.84	3.48±5.16	-2.74±5.08	-1.707	0.122
OAR					
Spinal cord Maximum dose	2383.08±353.89	3181.48±556.33	-798.40±303.74	-8.312	0.001
Esophagus					
V_50_ (%)	17.79±14.08	14.68±13.39	4.80±7.56	2.007	0.076
Maximum dose	5270.17±109.33	5205.15±96.40	-341.53±120.23	-0.898	0.392
Heart					
V_20_ (%)	20.78±21.65	20.76±21.46	-6.78±8.27	-2.591	0.029
V_30_ (%)	9.83±10.15	13.12±15.14	-6.46±9.26	-2.205	0.055
V_40_ (%)	4.87±5.24	5.32±5.49	-1.54±2.34	-2.090	0.066
V_45_ (%)	3.59±3.84	3.86±4.20	-0.96±1.81	-1.676	0.128
Mean dose	932.07±702.92	983.30±742.64	-284.71±339.66	-2.651	0.026
Normal lung					
V_5_ (%)	49.59±6.17	46.88±7.19	9.18±2.45	11.829	0.001
V_10_ (%)	35.13±7.42	31.74±6.64	4.85±4.05	3.791	0.004
V_13_ (%)	30.31±6.38	24.71±4.66	4.49±2.86	4.954	0.001
V_20_ (%)	22.22±4.48	17.95±2.56	1.54±1.52	3.185	0.011
V_30_ (%)	8.63±1.47	11.34±2.11	-2.49±2.09	-3.761	0.004
Mean dose	1040.41±147.82	1001.52±127.12	-29.12±286.09	-0.322	0.755
B-P					
V_5_(%)	26.15±3.14	20.49±2.71	5.66±1.76	10.143	0.001
V_10_(%)	16.54±2.41	14.32±2.43	2.23±1.03	6.824	0.001
V_15_(%)	10.97±1.80	11.92±3.92	-0.94±3.24	-0.920	0.382
V_20_(%)	6.46±1.43	10.04±3.22	-3.58±2.41	-4.684	0.001
V_25_(%)	3.72±0.93	8.32±2.76	-4.60±2.19	-6.648	0.001
V_30_(%)	2.13±0.55	5.76±2.33	-3.63±-1.99	-5.754	0.001
V_35_(%)	1.22±0.29	3.27±1.80	-2.05±1.65	-3.921	0.004
V_40_(%)	0.66±0.14	1.72±1.46	-1.05±1.39	-2.397	0.040
V_45_(%)	0.25±0.04	0.87±1.03	-0.62±1.02	-2.136	0.049
V_50_(%)	0.00±0.0	0.06±0.06	-0.05±0.05	10.143	0.007

**Table 10:** Comparisons of PTV, OAR, and B-P dosimetric parameters between ARC plan and CRT+ARC plan $\left(cGy,\overset{-}{x}\pm s\right)$.

Dosimetric parameters	ARC plan	CRT+ARC plan	Difference	*t *value	*P *value
PTV					
Minimum dose/cGy	4285.12±159.09	4305.23±114.48	-20.11±157.12	-0.405	0.695
Maximum dose/cGy	5348.30±61.22	5328.32±58.00	19.98±37.33	1.692	0.125
Median dose/cGy)	5025.60±6.27	5068.44±77.68	-42.84±79.30	-1.708	0.122
Conformity index	0.88±0.02	0.86±0.02	0.01±0.01	3.500	0.007
Homogeneity index	0.21±0.04	0.20±0.03	0.01±0.03	0.633	0.543
V_105_ (%)	0.74±.84	3.75±9.42	-3.01±9.58	-0.993	0.347
OAR					
Spinal cord Maximum dose	2383.08±353.89	2870.81±535.92	-487.73±224.92	-6.857	0.001
Esophagus					
V_50_ (%)	17.79±14.08	17.02±12.75	2.45±5.72	1.358	0.207
Maximum dose	5270.17±109.33	5229.34±79.93	-365.72±383.31	-0.954	0.365
Heart					
V_20_ (%)	20.78±21.65	20.56±21.20	-6.57±8.02	-2.592	0.029
V_30_ (%)	9.83±10.15	14.39±13.89	-7.72±7.93	-3.080	0.013
V_40_ (%)	4.87±5.24	4.88±4.73	-1.09±1.58	-2.193	0.056
V_45_ (%)	3.59±3.84	2.94±2.42	-0.04±0.19	-0.734	0.481
Mean dose	932.07±702.92	872.64±788.90	-174.05±470.84	-1.169	0.272
Normal lung					
V_5_ (%)	49.59±6.17	48.77±9.22	7.30±5.31	4.346	0.002
V_10_ (%)	35.13±7.42	28.30±5.43	8.30±2.97	8.832	0.001
V_13_ (%)	30.31±6.38	22.40±4.28	6.79±3.67	5.856	0.001
V_20_ (%)	22.22±4.48	14.74±2.08	4.75±2.39	6.281	0.001
V_30_ (%)	8.63±1.47	10.36±0.81	-1.50±1.06	-4.461	0.002
Mean dose	1040.41±147.82	952.15±120.92	20.24±91.13	0.222	0.829
B-P					
V_5_(%)	26.15±3.14	25.22±12.40	0.93±11.79	0.250	0.808
V_10_(%)	16.54±2.41	14.86±6.45	1.68±6.01	0.885	0.399
V_15_(%)	10.97±1.80	10.13±4.22	0.85±3.89	0.692	0.506
V_20_(%)	6.46±1.43	7.99±2.65	-1.53±2.15	-2.250	0.051
V_25_(%)	3.72±0.93	6.83±1.99	-3.11±1.51	-6.512	0.001
V_30_(%)	2.13±0.55	5.33±1.80	-3.19±1.51	-6.645	0.001
V_35_(%)	1.22±0.29	3.42±1.89	-2.19±1.74	-3.973	0.003
V_40_(%)	0.66±0.14	1.58±1.71	-0.92±1.65	-1.766	0.111
V_45_(%)	0.25±0.04	0.68±1.19	-0.43±1.18	-1.147	0.281
V_50_(%)	0.00±0.0	0.12±0.33	-0.11±0.32	-1.105	0.298

**Table 11:** Comparisons of PTV, OAR, and B-P dosimetric parameters between CRT+IMRT plan and CRT+ARC plan $\left(cGy,\overset{-}{x}\pm s\right)$.

Dosimetric parameters	CRT+IMRT plan	CRT+ARC plan	Difference	*t *value	*P *value
PTV					
Minimum dose/cGy	4051.12±183.68	4305.23±114.48	-254.11±132.76	-6.053	0.001
Maximum dose/cGy	5373.66±103.43	5328.32±58.00	45.34±115.42	1.242	0.246
Median dose/cGy)	5017.78±9.25	5068.44±77.68	-50.66±74.52	-2.150	0.060
Conformity index	0.80±0.03	0.86±0.02	0.07±0.03	-7.334	0.001
Homogeneity index	0.27±0.05	0.20±0.03	0.06±0.03	5.364	0.001
V_105_ (%)	3.48±5.16	3.75±9.42	-0.27±11.52	-0.074	0.943
OAR					
Spinal cord Maximum dose	3181.48±556.33	2870.81±535.92	310.67±262.39	3.744	0.005
Esophagus					
V_50_ (%)	14.68±13.39	17.02±12.75	-2.34±4.33	-1.709	0.122
Maximum dose	5205.15±96.40	5229.34±79.93	-24.19±73.44	-1.042	0.325
Heart					
V_20_ (%)	20.76±21.46	20.56±21.20	0.20±0.53	1.178	0.269
V_30_ (%)	13.12±15.14	14.39±13.89	-1.27±2.18	-1.843	0.098
V_40_ (%)	5.32±5.49	4.88±4.73	0.45±0.88	1.617	0.140
V_45_ (%)	3.86±4.20	2.94±2.42	0.92±1.88	1.547	0.156
Mean dose	983.30±742.64	872.64±788.90	110.66±322.92	1.084	0.307
Normal lung					
V_5_ (%)	46.88±7.19	48.77±9.22	-1.88±5.28	-1.127	0.289
V_10_ (%)	31.74±6.64	28.30±5.43	3.44±3.07	3.546	0.006
V_13_ (%)	24.71±4.66	22.40±4.28	2.31±2.17	3.376	0.008
V_20_ (%)	17.95±2.56	14.74±2.08	3.22±1.45	7.026	0.001
V_30_ (%)	11.34±2.11	10.36±0.81	0.99±2.04	1.528	0.161
Mean dose	1001.52±127.12	952.15±120.92	49.37±30.51	5.117	0.001
B-P					
V_5_(%)	20.49±2.71	25.22±12.40	-4.73±11.15	-1.341	0.213
V_10_(%)	14.32±2.43	14.86±6.45	-0.54±5.74	-0.300	0.771
V_15_(%)	11.92±3.92	10.13±4.22	1.79±4.96	1.143	0.283
V_20_(%)	10.04±3.22	7.99±2.65	2.05±3.37	1.923	0.087
V_25_(%)	8.32±2.76	6.83±1.99	1.49±2.74	1.715	0.120
V_30_(%)	5.76±2.33	5.33±1.80	0.43±2.47	0.553	0.594
V_35_(%)	3.27±1.80	3.42±1.89	-0.15±2.56	-0.186	0.856
V_40_(%)	1.72±1.46	1.58±1.71	0.13±2.19	0.195	0.850
V_45_(%)	0.87±1.03	0.68±1.19	0.19±1.57	0.393	0.703
V_50_(%)	0.06±0.06	0.12±0.33	-0.06±0.32	-0.553	0.594

## Data Availability

The data used to support the findings of this study are available from the corresponding author upon request.

## References

[1] Spira A., Ettinger D. S. (2004). Multidisciplinary management of lung cancer. *The New England Journal of Medicine*.

[2] Burdett S., Parmar M. K. B., Stewart L. A., Souhami R. L. (1998). Postoperative radiotherapy in non-small-cell lung cancer: systematic review and meta-analysis of individual patient data nine randomized controlled trials. PORT meta-analysis trialists group. *Lancet*.

[3] Lally B. E., Zeltennan D., Colasanto J. M., Haffty B. G., Detterbeck F. C., Wilson L. D. (2006). Postoperative radiotherapy for stage II or III non-small-cell lung cancer using the surveillance, epidemiology, and end results database. *Journal of clinical oncology*.

[4] Douillard J.-Y., Rosell R., De Lena M., Riggi M., Hurteloup P., Mahe M.-A. (2008). Impact of postoperative radiation therapy on survival in patients with complete resection and stage I, II, or IIIA non-small-cell lung cancer treated with adjuvant chemotherapy: the adjuvant navelbine international trialist association (ANITA) randomized trial. *International Journal of Radiation Oncology • Biology • Physics*.

[5] Graeter T. P., Sebastian B., Bischof M., Kugler G., Fischer J. R., Schneider T. (2016). Analysis of patients unable to undergo adjuvant chemotherapy after surgery in stage IIIA/B non-small cell lung cancer: will they benefit from mediastinal radiotherapy?. *The Thoracic and Cardiovascular Surgeon*.

[6] Feng W., Zhang Q., Fu X.-L. (2015). The emerging outcome of postoperative radiotherapy for stage IIIA(N2) non-small cell lung cancer patients: based on the three-dimensional conformal radiotherapy technique and institutional standard clinical target volume. *BMC Cancer*.

[7] Ohguri T., Yahara K., Moon S. D. (2012). Postoperative radiotherapy for incompletely resected non-small cell lung cancer: Clinical outcomes and prognostic value of the histological subtype. *Journal of Radiation Research*.

[8] Kepka L., Bujko K., Orlowski T. M. (2011). Cardiopulmonary morbidity and quality of life in non-small cell lung cancer patients treated with or without postoperative radiotherapy. *Radiotherapy & Oncology*.

[9] Berman A. T., Teo B.-K. K., Dolney D. (2013). An in-silico comparison of proton beam and IMRT for postoperative radiotherapy in completely resected stage IIIA non-small cell lung cancer. *Journal of Radiation Oncology*.

[10] Remick J. S., Schonewolf C., Gabriel P. (2017). First clinical report of proton beam therapy for postoperative radiotherapy for non–small-cell lung cancer. *Clinical Lung Cancer*.

[11] Wang H.-H., Deng L., Wen Q.-L. (2017). Early postoperative radiotherapy is associated with improved outcomes over late postoperative radiotherapy in the management of completely resected (R0) stage IIIA-N2 nonsmall cell lung cancer. *Oncotarget*.

[12] Blom G. J., Verbakel W. F. A. R., Dahele M. A. X., Hoffmans D., Slotman B. J., Senan S. (2015). Improving radiotherapy planning for large volume lung cancer: a dosimetric comparison between hybrid-IMRT and RapidArc. *Acta Oncologica*.

[13] Agapito J. (2013). On the possible benefits of a hybrid VMAT technique in the treatment of non-small cell lung cancer. *Medical Dosimetry*.

[14] Jo I.-Y., Kay C.-S., Kim J.-Y. (2014). Significance of low-dose radiation distribution in development of radiation pneumonitis after helical-tomotherapy-based hypofractionated radiotherapy for pulmonary metastases. *Journal of Radiation Research*.

[15] Khalil A. A., Hoffmann L., Moeller D. S., Farr K. P., Knap M. M. (2015). New dose constraint reduces radiation-induced fatal pneumonitis in locally advanced non-small cell lung cancer patients treated with intensity-modulated radiotherapy. *Acta Oncologica*.

[16] Martin S., O'Brien R., Hofmann C., Keall P., Kipriditis J. (2018). An in silico performance characterization of respiratory motion guided 4DCT for high-quality low-dose lung cancer imaging. *Physics and Medicine and Biology*.

[17] Chamberland M., McEwen M. R., Xu T. (2016). Technical aspects of real time positron emission tracking for gated radiotherapy. *Medical Physics*.

[18] Ma C., Hou Y., Li H. (2014). A study of the anatomic changes and dosimetric consequences in adaptive crt of non-small-cell lung cancer using deformable CT and CBCT image registration. *Technology in Cancer Research & Treatment*.

[19] Cazzato R. L., Battistuzzi J.-B., Catena V. (2015). Cone-beam computed tomography (CBCT) versus CT in lung ablation procedure: which is faster?. *CardioVascular and Interventional Radiology*.

[20] Arns A., Blessing M., Fleckenstein J., Stsepankou D. (2016). Towards clinical implementation of ultrafast combined kV-MV CBCT for IGRT of lung cancer. *Strahlentherapie und Onkologie*.

